# A Blockchain-Enhanced Neural Network Framework for secure e-waste forecasting in smart cities

**DOI:** 10.3389/fpubh.2026.1779806

**Published:** 2026-04-28

**Authors:** Jussen Facuy, Ariel Pasini, Elsa Estévez, Cesar Moran, Wilmer Illescas, Verónica Veloz

**Affiliations:** 1 Universidad Agraria del Ecuador, Guayaquil, Guayas, Ecuador; 2 Instituto de Investigación en Informática III–LIDI, Facultad de Informática, Universidad Nacional de La Plata (UNLP), La Plata, Buenos Aires, Argentina; 3 Laboratorio de Ingeniería de Software y Sistemas de Información (LISSI), Departamento de Ciencias e Ingeniería de la Computación, Universidad Nacional del Sur (UNS), Bahía Blanca, Buenos Aires, Argentina; 4 Universidad Técnica de Machala, Machala, El Oro, Ecuador; 5 Universidad Estatal de Bolívar, Guaranda, Bolívar, Ecuador

**Keywords:** blockchain, electronic waste forecasting, neural networks, secure data governance, smart cities

## Abstract

The exponential growth of electronic waste (e-waste) represents one of the most critical environmental challenges faced by contemporary urban systems. Accurate forecasting of e-waste generation is essential for supporting sustainable decision-making within smart cities. This study proposes the Blockchain-Enhanced Neural Network Framework (BENNF), a conceptual and architectural framework designed to enable secure, transparent, and scalable e-waste forecasting through the integration of artificial intelligence, big data analytics, and blockchain technology. The proposed framework is structured into three interconnected layers: (i) a data acquisition and preprocessing layer based on big data pipelines (Apache Spark and Hadoop); (ii) a prediction layer employing multilayer neural networks trained on socioeconomic and environmental variables; and (iii) a blockchain layer that ensures data integrity, transparency, and traceability through smart contracts and a Proof-of-Authority consensus mechanism. Unlike fully deployed empirical systems, BENNF is presented as a system-level and design-oriented framework, aimed at strengthening digital governance and trust in data-driven environmental management. The framework aligns with the Sustainable Development Goals (SDGs) 12 and 13, promoting responsible consumption, circular economy principles, and climate-resilient urban planning. Its potential applicability in urban contexts such as Guayaquil, Ecuador, highlights its scalability and relevance for sustainable smart city initiatives.

## 1 Introduction

According to recent global reports, the generation of electronic waste has increased significantly due to rapid technological consumption and shorter device life cycles (1). Furthermore, consumer behavior related to electronic waste recycling remains a critical challenge for sustainable waste management systems (2). This exponential growth reflects the impact of accelerated technological consumption and the shortening of electronic device life cycles WEEE contains valuable materials such as gold, copper, and lithium, but also toxic components that, without proper management, threaten ecosystems and human health (3, 4). Therefore, accurate prediction of its generation has become an imperative need to plan sustainable policies and strengthen the circular economy within the framework of sustainable smart cities (5). Smart cities integrate digital infrastructure, sensors, and data systems that enable efficient resource management (6, 7). In this context, Ambient Intelligence (AmI) has emerged as a paradigm that seeks to adapt technology to both environmental and user needs, promoting sustainable urban development (8). However, the growing volume of data generated in these environments poses challenges for prediction, storage, and information security (9, 10). The generation of WEEE, as an indicator of urban technological behavior, becomes an essential variable for assessing the digital sustainability of cities (11).

Furthermore, the effects of climate change intensify pressure on urban and technological systems. Recent studies, such as “Impacts of Climate Change on Extreme Weather Indices in Ecuadorian Cities: A Socioeconomic Analysis,” demonstrate that extreme climatic fluctuations affect both infrastructure and energy consumption patterns for cooling, leading to higher technological disposal (12). This link between climate variability and waste generation highlights the urgency of developing comprehensive predictive models capable of incorporating environmental and socioeconomic variables into intelligent WEEE management systems, thus strengthening urban resilience and technological sustainability (13).

Several recent studies have applied Machine Learning (ML) techniques to estimate WEEE quantities and optimize environmental planning (14, 15). Predictive models such as Artificial Neural Networks (ANN), Decision Trees, and boosting methods (e.g., XGBoost) have demonstrated high accuracy in modeling complex environmental data (16, 17). Along these lines, Facuy et al. (14) developed a predictive model based on Ambient Intelligence, focused on electronic waste generation from the perspective of sustainable smart cities. Subsequently, they expanded their approach by validating an XGBoost model applied to the urban context of Guayaquil, Ecuador, demonstrating the effectiveness of statistical learning for quantitative WEEE estimation (15). Likewise, their neural network–based predictive model emphasized the relevance of Ambient Intelligence as an integrating axis of sustainability, technology, and urban management (16).

Nevertheless, although these approaches have improved WEEE prediction accuracy, challenges persist in terms of data security, traceability, and reliability (18). Current systems often rely on centralized data architectures, making them vulnerable to manipulation, cyberattacks, or loss of data integrity (19). In addition, the management of large volumes of heterogeneous data (Big Data) from sources such as IoT sensors, recycling platforms, and municipal records requires transparent and auditable mechanisms to ensure data veracity throughout the information flow (20, 21).

In this context, blockchain technology has emerged as a disruptive tool to strengthen data security and traceability in smart urban environments (22, 23). Its decentralized structure, based on consensus and cryptography, allows the storage of immutable and verifiable records, which is especially useful in WEEE management (24, 25). In combination with Deep Learning, blockchain can create a trusted environment that ensures the integrity of predictive models and the data used in them (26, 27). For instance, recent studies have proposed blockchain use in the electronic recycling chain to track devices from manufacturing to final disposal (28). Others have integrated blockchain with Artificial Intelligence to optimize data collection in urban waste management systems (29, 30).

This study proposes a conceptual framework called the Blockchain-Enhanced Neural Network Framework (BENNF), aimed at the secure and sustainable prediction of electronic waste in smart cities (31). This approach is based on the interconnection of three pillars: (i) Ambient Intelligence, which provides an environment sensitive and adaptable to urban sustainability (32); (ii) Neural Networks, which enhance predictive capability through learning complex patterns (33); and (iii) Blockchain technology, which guarantees the security, transparency, and traceability of the data employed. Together, these elements form an architecture capable of enhancing Big Data analytics for predictive and sustainable purposes (34, 35).

The use of blockchain not only provides security but also resilience against attacks and verifiable auditing of AI models, representing a qualitative leap compared to traditional data architectures (36, 37). Such hybrid frameworks align with Sustainable Development Goal (SDG) 12 (Responsible Production and Consumption) and the 2030 Agenda, promoting technological innovation to reduce waste and efficiently use resources (38, 39). They also strengthen digital governance and transparency in public environmental management policies (40, 41).

In the scientific field, the convergence of Blockchain, Big Data, and Artificial Intelligence has been recognized as one of the most promising technological trends for urban sustainability (42, 43). Recent research emphasizes that combining these approaches can transform environmental prediction and control systems by enabling decision-making based on verifiable data and robust models (44). However, there is still a conceptual gap limiting the integrated application of these technologies in WEEE management (45). Most current studies address these tools independently, without establishing a synergistic framework that combines security, predictive capability, and sustainability (46).

This article contributes to closing this gap by formulating a secure and scalable theoretical framework for WEEE prediction in smart cities. Unlike previous approaches, it proposes a holistic integration that combines the transparency and traceability of blockchain with the predictive power of neural networks within the Ambient Intelligence ecosystem. This model aims to serve as a foundation for future developments that enable secure, decentralized prediction systems aligned with urban sustainability principles (47).

Despite significant advances in machine learning approaches for electronic waste prediction, most existing studies focus primarily on predictive accuracy while overlooking issues related to data security, transparency, and traceability. In addition, many current solutions rely on centralized infrastructures that may expose sensitive data to potential manipulation. This limitation highlights the need for integrated frameworks that combine predictive analytics with decentralized technologies. The proposed Blockchain-Enhanced Neural Network Framework (BENNF) addresses this research gap by integrating neural network prediction with blockchain-based validation mechanisms to ensure secure and transparent e-waste data management.

The primary contribution of this work lies in the design and integration of a secure system architecture, rather than in the empirical benchmarking of predictive models. The proposed framework aims to provide a robust conceptual foundation for future pilot implementations and data-driven governance strategies in smart city environments.

In terms of organization, this paper is structured as follows: Section II presents the theoretical foundations and review of related works; Section III describes the proposed conceptual architecture of the blockchain–neural network framework; Section IV discusses the implications, challenges, and implementation opportunities in the context of sustainable smart cities; and finally, Section V presents the conclusions and future research directions.

## 2 Theoretical framework and related work

The integration of Artificial Intelligence (AI), Big Data, and Blockchain technology has driven the development of intelligent and secure infrastructures aimed at environmental management within smart cities. These technologies enable the automation of data collection, storage, and analysis, strengthening predictive capabilities while ensuring transparency in urban sustainability systems (23, 31, 35).

To contextualize the contribution of the proposed framework, Table 1 presents a comparative overview of existing approaches for electronic waste prediction and management. The comparison highlights the limitations of traditional statistical models, machine learning approaches, and blockchain-based waste tracking systems. Unlike previous methods, the proposed Blockchain-Enhanced Neural Network Framework (BENNF) integrates predictive intelligence with secure decentralized data governance.

**Table 1:** Comparison of AI-based approaches for e-waste prediction.

Model	Technology used	Security mechanism	Prediction capability	Limitations
Traditional statistical models	Linear Regression	None	Low for complex data	Cannot model nonlinear relationships
Machine learning models	Random Forest, XGBoost	Centralized storage	Moderate accuracy	Vulnerable to data manipulation
Deep learning models	ANN, CNN, RNN	Centralized databases	High predictive power	Lack transparency and traceability
Blockchain waste tracking systems	Blockchain + IoT	Distributed ledger	Waste tracking only	Limited predictive capabilities
Proposed BENNF framework	Neural Networks + Blockchain + Big Data	Smart contracts + Proof-of-Authority	High predictive accuracy + secure data governance	Conceptual architecture pending full deployment

### 2.1 Neural networks and artificial intelligence for electronic waste prediction

Neural Networks (NN) have been widely applied in environmental forecasting tasks due to their ability to model nonlinear and multivariate relationships (16, 33). In addition, comparative studies evaluating multiple machine learning algorithms have demonstrated that hybrid and ensemble models can significantly improve prediction accuracy for electronic waste generation. These studies highlight the relevance of combining deep learning architectures with traditional machine learning techniques to capture complex patterns in environmental and socioeconomic data (13). In the field of electronic waste prediction, deep architectures such as Multilayer Perceptrons (MLP), Convolutional Neural Networks (CNN), and Recurrent Neural Networks (RNN) have shown significant performance in learning temporal and socioeconomic patterns that influence e-waste generation (13, 17).

In particular, hybrid models that combine statistical learning with deep learning, such as the integration of XGBoost with Artificial Neural Networks, have proven effective in capturing dependencies among heterogeneous variables. These approaches improve prediction accuracy by leveraging both structured data (socioeconomic indicators) and unstructured data (textual or sensor data) (14, 15).

### 2.2 Blockchain for secure and transparent data governance

Blockchain provides a decentralized, immutable, and verifiable system for information storage (22, 24, 36). In waste management, this technology ensures traceability and transparency throughout the life cycle of electronic products, from production to final disposal, preventing manipulation or data loss (25, 28).

Each transaction within the distributed ledger represents an auditable record in real time, increasing accountability among the actors involved: manufacturers, recyclers, governmental institutions, and consumers. This approach is particularly relevant in smart cities, where multiple IoT systems and data platforms continuously interact (29, 43).

### 2.3 Integration between blockchain and neural networks

The convergence of Blockchain and AI, particularly neural networks, enables the creation of secure analytical ecosystems where data integrity and predictive intelligence coexist (26, 37). Blockchain guarantees the reliability of datasets used by neural networks, reducing risks of bias or corruption during model training and validation.

On the other hand, AI enhances Blockchain performance through automated anomaly detection, optimization of consensus mechanisms, and prediction of failures or overloads in network nodes (31, 44). This bidirectional relationship supports the development of trustworthy and auditable AI systems, especially in environmental applications where transparency is critical (42, 45).

## 3 Proposed architecture: Blockchain-Enhanced Neural Network Framework (BENNF)

**Figure 1:**
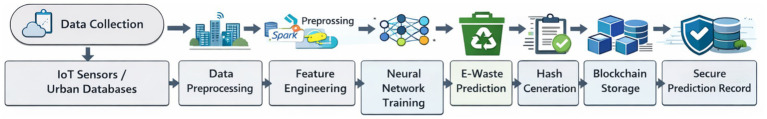
Technical workflow of the blockchain-AI integration. Technical workflow illustrating the integration of Artificial Intelligence and blockchain for predicting e-waste generation in smart city ecosystems.

The proposed model, called the Blockchain-Enhanced Neural Network Framework (BENNF), aims to ensure the secure, transparent, and scalable prediction of electronic waste generation in smart urban contexts. The architecture consists of three main layers (Figure 1):

1 Data acquisition and preprocessing layer
2 Neural network–based prediction layer

To provide a clearer understanding of the interaction between artificial intelligence and blockchain technologies within the proposed framework, Figure 1 presents the technical workflow of the BENNF system. The diagram illustrates the sequence of processes, including data acquisition from urban sources, big data preprocessing, neural network-based prediction, and blockchain validation of forecasting results.

Technical workflow of the Blockchain-Enhanced Neural Network Framework (BENNF) integrating data processing, neural network prediction, and blockchain validation.

3 Blockchain security and traceability layer

**Figure 2:**
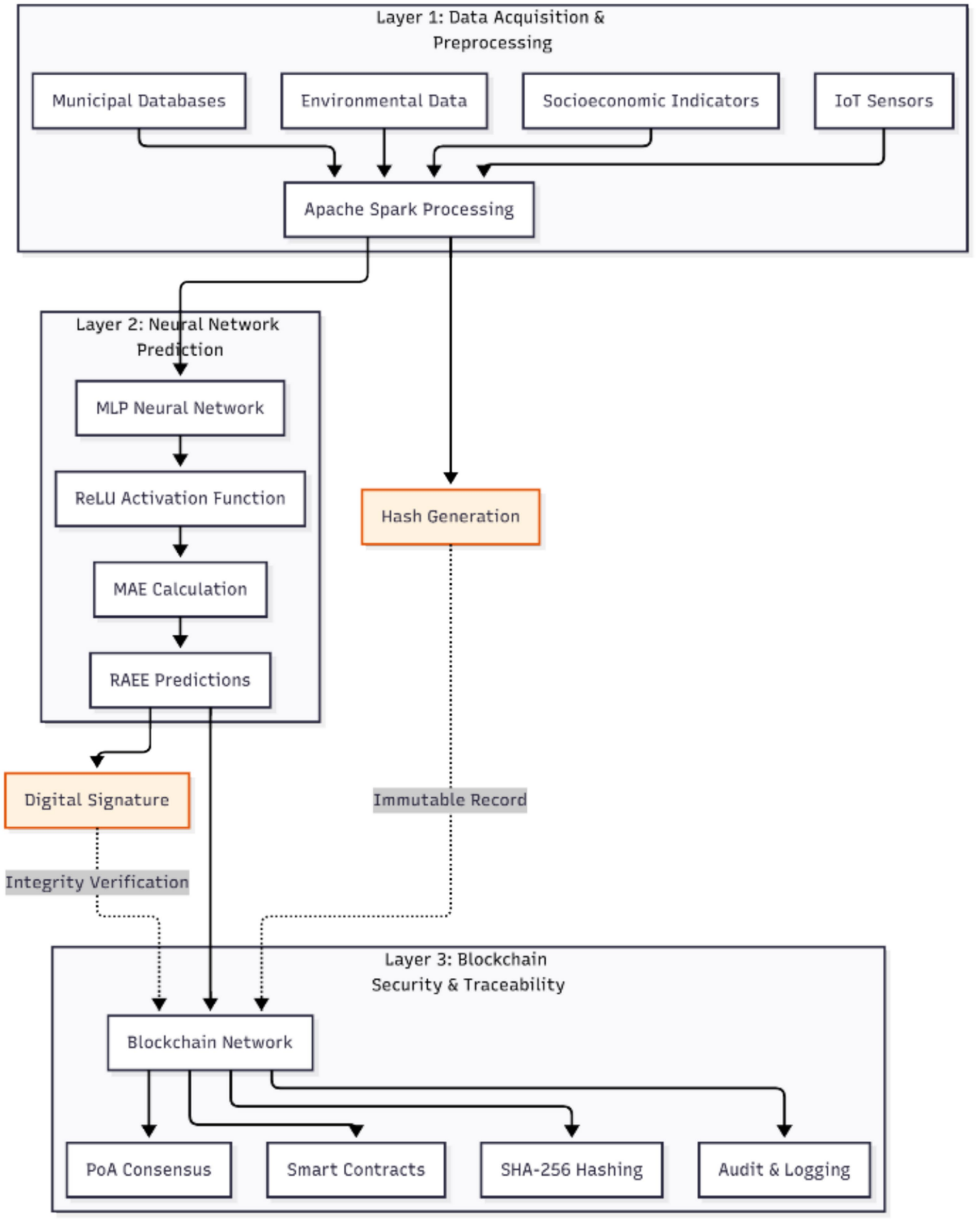
Three-layer BENNF architecture for secure e-waste prediction.

Figure 2 illustrates the three-layer architecture of the Blockchain-Enhanced Neural Network Framework (BENNF), including data acquisition and preprocessing, neural network-based prediction, and blockchain-based security and traceability.

### 3.1 Data acquisition and preprocessing layer

This layer integrates and processes various sources of information, including:

1 IoT sensors recording rates of electronic device disposal
2 Municipal and recycling center databases
3 Socioeconomic indicators (income level, consumption, education, urban density)
4 Environmental variables (temperature, humidity, climate indices)

Processing is performed through Big Data channels based on Apache Spark and Hadoop, enabling real-time management of large volumes of information. Data are cleaned, structured, and encoded for neural network training. Metadata from these datasets are stored in Blockchain nodes to ensure the integrity and authenticity of data sources (20, 30).

### 3.2 Neural network–based prediction layer

The predictive core of the system consists of a multilayer neural network (MLP) trained using a hybrid approach that combines statistical optimization and deep learning. Mathematically, the network can be represented as:


$$Y=f(W_{2}\cdot \sigma (W_{1}X+b_{1})+b_{2})$$

where X represents the input vector of socioeconomic and environmental variables, W and b denote the weights and biases, and σ(·) corresponds to the ReLU activation function.

The minimization of the prediction error is carried out using the Mean Absolute Error (MAE) function:


$$\mathrm{MAE}=\frac{1}{n}\sum_{i=1}^{n}|y_{i}-{\hat{y}}_{i}|.$$

Once trained, the model predicts the expected amount of electronic waste across different periods and geographical areas. The results are cryptographically signed and stored on the blockchain, guaranteeing their immutability and verifiability.

### 3.3 Blockchain security and traceability layer

This layer ensures transparency, decentralization, and traceability of the data flow. Each transaction, from initial collection to final prediction, is recorded using (smart contracts) that establish:

- Validation rules for incoming data
- Access permissions to the AI model
- Auditing mechanisms for the stakeholders involved

The blockchain operates under a Proof-of-Authority (PoA) consensus mechanism, providing high security with low energy consumption (36). Additionally, Application Programming Interfaces (APIs) ensure interoperability between the AI environment and the blockchain network.

## 4 Discussion and implementation perspectives

The Blockchain-Enhanced Neural Network Framework (BENNF) represents a significant advancement at the intersection of Artificial Intelligence (AI), blockchain, and urban sustainability, offering a comprehensive approach for the se-cure prediction and management of electronic waste (e-waste) within sustainable smart cities. This model not only addresses the environmental challenges posed by the exponential growth of e-waste, projected to exceed 82 million tons by 2030 (1, 3), but also introduces a reliable and auditable digital architecture that is essential for decentralized governance environments.

The BENNF integrates three strategic axes:

1 Predictive accuracy, achieved through deep neural networks capable of processing heterogeneous and multiscale data from IoT sensors, urban collection platforms, and recycling systems (6, 13, 30);
2 Integrity and traceability of information, ensured by cryptographic mechanisms and distributed consensus intrinsic to blockchain technology (24, 25, 47);
3 Transparency and data governance, through the use of auditable smart contracts and interoperability protocols for public administrations (20, 38, 40).

From an operational perspective, this framework can be integrated into intelligent urban control dashboards, enabling municipal and environmental authorities to anticipate waste generation trends, optimize collection logistics, and design evidence-based recycling policies (5, 7, 21). Moreover, its modular architecture facilitates expansion toward other sustainability dimensions, such as the prediction of pollutant emissions, urban energy consumption, and water-use efficiency (12, 33, 35).

### 4.1 Pilot implementation scenario

Luego pega tu texto (ligeramente integrado para que fluya mejor en el artículo):

To strengthen the practical applicability of the proposed framework, a pilot implementation of the Blockchain-Enhanced Neural Network Framework (BENNF) could be deployed in a smart city environment such as Guayaquil, Ecuador.

In this scenario, data sources including municipal waste management databases, recycling centers, IoT-enabled smart waste containers, and socioeconomic indicators could be integrated through a Big Data infrastructure supported by platforms such as Apache Spark and Hadoop.

The neural network model would be trained using historical datasets related to electronic waste generation, urban population growth, technology consumption patterns, and environmental variables.

Blockchain nodes operated by municipal institutions, environmental agencies, and recycling organizations would validate and record prediction results through smart contracts, ensuring transparency and data integrity.

This pilot deployment would allow the evaluation of BENNF in terms of prediction accuracy, computational scalability, system latency, and governance transparency within a real smart city ecosystem.

From a technical standpoint, the integration of blockchain and neural networks allows the establishment of hybrid blockchains where data preprocessed by AI nodes are cryptographically validated before storage. This reduces vulnerability to manipulation and ensures information authenticity throughout the entire electronic waste life cycle (23, 29, 42). The combination of Explainable Machine Learning (XAI) and blockchain can further enhance prediction interpretability and enable algorithmic auditing in regulatory processes (19, 37, 43).

In the context of smart cities, where digital infrastructures, urban sensors, and open data platforms converge, the BENNF acts as a trust nexus between predictive analytics and environmental cybersecurity.

This symbiotic relationship drives a model of Ambient Intelligence (AmI) that, according to Facuy and Espinoza (8), promotes sustainability grounded in ecological awareness and informed decision-making. At the urban level, this translates into the circular optimization of material flows, aligned with resilience and green economy goals outlined in the 2030 Agenda (39).

Despite its potential, the real-world deployment of BENNF faces several key challenges that must be addressed for successful implementation:

- Computational scalability: The simultaneous execution of deep learning algorithms and blockchain validations demands high computational capacity and energy consumption. This calls for energy-efficient strategies such as the use of federated networks and edge computing to distribute processing loads (6, 35).
- Data heterogeneity and interoperability: Current urban systems produce fragmented data. Open standards and common ontological models are required to integrate information from diverse sources such as IoT devices, satellites, municipal records, and recycling platforms (7, 10, 42).
- Cybersecurity and privacy: The decentralized management of environmental information necessitates robust mechanisms for anonymization, homomorphic encryption, and fault-tolerant consensus protocols to prevent data breaches (18, 19, 47).
- Ethical and regulatory aspects: It is essential to define responsibilities regarding data ownership and usage, citizen access rights, and algorithmic auditing protocols to ensure fair and transparent information governance (20, 41, 43).

From a sustainability standpoint, the application of BENNF contributes to strengthening the digital infrastructure of smart cities, promoting circularity in waste management and citizen participation based on verifiable data (5, 38, 45). Its adoption can also serve as a replicable model in Global South countries, where digitalization and sustainability challenges are more pronounced (21, 38).

Recent literature shows increasing interest in the convergence of AI and blockchain for sustainable urban development. For example, Razaque et al. (19) emphasize the value of multilayer cybersecurity architectures, while Nallagattla et al. (35) and Jiang et al. (29) propose energy-efficient and digital governance approaches to minimize the en-vironmental footprint of intensive computation.

These findings reinforce the relevance of BENNF as a technologically viable and environmentally responsible model. In the future, the line of research could be oriented in three main directions:

1 Integration of explainable hybrid models, combining deep learning with interpretability techniques based on fuzzy logic and causality to ensure transparency in automated decision-making (37, 43).
2 Development of sustainable data public policies, supported by digital governance mechanisms such as dynamic smart contracts that adjust traceability rules according to environmental indicators (20, 40, 44).
3 Energy optimization of BENNF, through the implementation of green algorithms and low-carbon consensus mechanisms, leveraging the potential of quantum and neuromorphic computing (35, 47).

In addition, BENNF not only provides a secure and verifiable prediction mechanism for electronic waste management but also introduces a new paradigm in digital urban governance, aligned with the vision of resilient, sustainable, and ethically managed smart cities. The combination of AI, blockchain, and ambient intelligence paves the way toward an urban future in which technology not only optimizes processes but also guarantees transparency, sustainability, and social trust.

## 5 Conclusion

This study presents a secure prediction framework based on Neural Networks and Blockchain (BENNF) aimed at the sustainable management of electronic waste in smart cities. The proposal integrates Big Data, Artificial Intelligence (AI), and Distributed Ledger Technologies (DLT) to ensure transparency, accuracy, and traceability of environmental information.

The model contributes directly to the achievement of Sustainable Development Goals (SDGs) 12 and 13, by fostering responsible production and climate resilience through digital innovation.

As future work, the following research and implementation lines are proposed:

1 Implement the BENNF in a pilot city (Guayaquil, Ecuador) to evaluate its scalability and efficiency
2 Incorporate quantum-resistant cryptographic algorithms.
3 Develop energy-efficient learning (Green AI) models that reduce the com-putational footprint and promote digital sustainability.

This interdisciplinary integration lays the foundation for a smart, safe, and sustainable urban ecosystem, capable of harnessing the power of data for responsible environmental management.

## Data Availability

The original contributions presented in the study are included in the article/supplementary material, further inquiries can be directed to the corresponding author.
